# Strength-based strategies for addressing racial stressors in African American families: lessons learned from developing the LEADS health promotion intervention

**DOI:** 10.1007/s10865-024-00509-y

**Published:** 2024-08-10

**Authors:** Timothy Simmons, Mary Quattlebaum, Pamela Martin, Dawn K. Wilson

**Affiliations:** Department of Psychology, University of South Carolina, Columbia, SC USA

**Keywords:** African American families, Stress, Physical activity, Community-based participatory research, Strength-based approaches, Intervention

## Abstract

Recognizing the real-life impact of racial stress on physical and psychological health is vital for creating impactful health promotion interventions among African American families. Despite the known link between racial stress and poor physical health outcomes, no existing intervention to date has targeted stress management strategies to buffer racial stress and build positive health behaviors among African American families. The current study outlines the lessons learned throughout the development of the Linking Exercise for Advancing Daily Stress (LEADS) Management and Resilience program, a 10-week family-based health promotion, stress management, and resilience intervention that aimed to improve physical activity, healthy eating, and well-being among African American adolescents and parents. We highlight the evolution of the LEADS intervention from a health promotion and stress management intervention to a culturally salient health promotion, stress management, and resilience intervention utilizing community-based participatory research strategies. This paper chronicles our systematic journey in making those changes and the lessons we learned along the way. We provide specific recommendations and implications for future health promotion interventions developed for African American families. Overall, we argue for a research orientation that respects cultural and racial contexts, embraces diversity within research teams and self-reflection, recognizes the heterogeneity among African American populations, and applies strength-based approaches.

**Supplementary Information:**

The online version contains supplementary material available at 10.1007/s10865-024-00509-y.

## Background

Racial stress, such as experiences of discrimination, prejudice, and bias based on one’s race, is recognized as one of the most prominent stressors for African American youth and their families (McNeil Smith & Landor, 2018). Literature suggests that experiences of racial stress and discrimination are identified as early as preschool and persist throughout one’s lifespan (Jones et al., 2020). It has been well-established that there is a strong relationship between racism and health (Williams et al., 2003). Specifically, exposure to racial stressors can significantly hinder psychological well-being (e.g., depression and anxiety), physical health-related outcomes (e.g., hypertension, inflammation), high risk health behaviors, and academic performance (Lozada et al., 2022; Banerjee et al., 2023; Williams & Mohammed, 2009). These chronic stressors and associated consequences may serve as a major barrier to engagement in health promotion efforts. Understanding the lived experiences of racial stress and health and identifying protective buffers to these stressors for African American families is important for developing effective and engaging health promotion interventions for these families.

Exposure to environmental and systemic stressors has been shown to create barriers to engaging in healthy behaviors that contribute to poor physical health behaviors and health outcomes among families from minority racial-ethnic backgrounds (Jackson et al., 1996). Recent research by our group has shown that numerous chronic stressors, such as racial discrimination, may undermine the engagement in and impact of family-based health promotion programs (Kipp et al., 2024; Quattlebaum et al., 2021), thus highlighting the critical need to address chronic stressors as part of family-based interventions. Specifically, ethnic-racial communities are at greater risk of having limited access to nutritious food options and safe spaces for physical activity (Williams & Mohammed, 2009). There is also evidence to suggest that institutional and structural racism is associated with decreased engagement in physical activity, due to lack of resources (Ahmed et al., 2007); however, some investigators have also shown mixed findings between racial stress and physical activity (Borrell et al., 2013). In addition, chronic household stress, which may include racial stressors, has been shown to be negatively correlated with the frequency of family meals (Fulkerson et al., 2019). Further, perceived racial stress has been associated with lower quality of dietary intake (energy-dense foods) among African American adolescents and adults (Cave et al., 2020). Despite this evidence, no existing intervention to date has targeted stress management strategies to buffer racial stress and build engagement in positive health behaviors (e.g., physical activity, health eating) among African American families.

This purpose of present paper is to highlight the evolution of the Linking Exercise for Advancing Daily Stress (LEADS) Management and Resilience Program in African American adolescents and their caregivers. The LEADS intervention is a health promotion and stress management intervention that integrates culturally salient health promotion, stress management, and resilience theories and which utilized community-based participatory research strategies. A growing literature suggests that leveraging family and cultural resilience resources inherently rooted within African American families may be a critical point of intervention to buffer the consequences of racial stress and for promoting positive mental health and academic outcomes. In fact, prior resilience-based interventions with African American youth and families have shown improvements across a broad range of outcomes related to psychological well-being, academic achievement, and risk-taking behaviors (Cave et al., 2020; Jackson et al., 1996; Lozada et al., 2022; Quattlebaum et al., 2024; Sellers et al., 2006; Wilson et al., 2021, 2022), but no such trial has targeted promoting engagement in health promoting behaviors such as physical activity. To address this literature gap, our team developed a health promotion program to explicitly address racial stressors (e.g., discrimination) to effectively improve engagement in positive health behaviors (physical activity, healthy eating) among African American families—the LEADS intervention. However, the development of the LEADS program was not without its challenges and there were critical lessons learned throughout the process. To highlight and advance the development and dissemination of such health promotion programs among African Americans, this paper outlines the process of developing the LEADS intervention, including lessons learned and recommendations for future programs.

## Lessons learned from the LEADS program

### Importance of utilizing participatory methods

“One falsehood spoils a thousand truths.”—African Proverb.

Historically, the lived experiences of Black individuals across the diaspora have been undervalued and misrepresented in research, and as the proverb goes, “one falsehood spoils a thousand truths,” these inaccuracies have profoundly skewed our understanding and perpetuated systemic harm in Black populations (Breland-Noble et al., 2024). More specifically, these falsehoods, based on pseudo-scientific theories over centuries, have led to deficit-oriented research that has perpetuated racist behaviors, policies, and laws (American Psychological Association, 2021; Hoffman et al., 2016; Holliday, 2009; Spettel & White, 2011; Spigner, 2007; Tettegah et al. 2023; Washington, 2007). Consistent with this African Proverb, community-based participatory research (CBPR) is essential to collaborating and developing an effective health promotion program targeting diverse communities. This research approach involves active participation from community members throughout the research process, from the initial planning stages to the dissemination of findings (Glasgow et al., 1999). Further, this approach recognizes that community members have valuable knowledge and insights that can contribute to the success of research projects. By involving community members in the research process, CBPR can help ensure that research is relevant, meaningful, and applicable to the community's needs and concerns (Glasgow et al., 1999). Further, this research process establishes a more collaborative, equitable, trusting relationship between community members and the researcher, which reduces power imbalances and promotes mutual respect (Israel et al., 1998; Wilson & Sweeney, 2024). This collaborative approach can lead to more reliable and valid data, greater engagement in the research process, and meaningful outcomes (Collins et al., 2018). CBPR has become increasingly important in developing programs to address health disparities and improve the health and well-being of underserved communities (Cyril et al., 2015; Wallerstein & Duran, 2010; Wilson & Sweeney, 2024).

Specifically, we adopted the seven CBPR principles created by the National Black Leadership Initiative on Cancer (NBLIC) for African American communities, which incorporate their unique contexts and traditions in research (Smith et al., 2015). These principles include: (1) underscoring the importance of the family; (2) creating trust between investigators, stakeholders, and the community to foster co-learning, collaborative decision-making, and shared ownership of the issues and their resolutions; (3) involving community partners as active participants in research building on their strengths and assets, as well as address areas needing enhancement; (4) appreciating the iterative process of research, which emphasizes reflection and revision, ensures that both researchers and communities are content with the results; (5) including community partners in the dissemination of the research; (6) engaging with community partners to comprehend the context and creating a collaborative strategy for implementing the intervention; and (7) exploring strategies with community partners to mitigate and eliminate the health disparities affecting African Americans (Smith et al., 2015). Thus, these principles played a key role in guiding the development of the LEADS program.

### Overview of the LEADs intervention program

The LEADS program involved a family-based online pilot intervention for overweight African American adolescents and their parents aimed to improve physical activity and stress management skills. The LEADS pilot trial was implemented between 2021 and 2023 and included 5 cohorts. Across the 5 cohorts, 33 African American parent-adolescent dyads participated (adolescents: ~ 61% female, age *M*(*SD*) 13.8 (2.06) years old, parents: ~ 97% female, age *M*(*SD*) 45.5 (10.30) years old; See also Table S1 Supplement Materials). The initial three cohorts compared a partially integrated cultural and family resilience health promotion intervention to a health education comparison group, whereas the final two cohorts compared a fully integrated cultural and family resilience health promotion intervention to the partially integrated cultural and family resilience health promotion intervention. A thorough overview of the curriculum differences between the partially integrated and fully integrated content is provided in Table S2 (see Supplemental Materials). The LEADS trial was 10-weeks in duration. Prior to starting the 10-week program, families attended a 2-week run-in period for orientation and addressing barriers to participation. Group sessions were held online once per week for 1.5 h. Group sizes for both the intervention and control group ranged from 5 to 6 families.

Throughout these 5 cohorts, our research team conducted in-depth process evaluation approaches to identify strengths and weaknesses of the program from the community’s perspective. With this feedback, we engaged in self-reflection as a team and opened our ears to participant’s voices and implemented their suggested changes. In line with the NBLIC’s CBPR principles, our research team along with previous participants and community partners identified four lessons that guided the development and adaptation made to the LEADS intervention over time. Specifically, we learned the importance of (1) developing a culturally sensitive curriculum; (2) recruiting diverse research team members with shared lived experiences and engaging in self-reflection; (3) recognizing heterogeneity among families from minority racial-ethnic backgrounds; and (4) utilizing a strength-based research approach. The comprehensive process in which we learned these lessons and adapted the LEADS program is described in detail below.

#### Lesson 1: developing a culturally sensitive curriculum

Initially, the LEADS intervention curriculum focused on health promotion and partially integrated cultural resilience and stress management content (See Table 1 for detailed Timeline). Weekly sessions included key content, interactive activities, group discussions, stress management techniques, group exercise videos, and family bonding take-home activities. Key content targeted a range of topics regarding health promotion, stress management, and positive parenting strategies (See Table S2 Supplemental Materials for curriculum details). As shown in Table 1 (Timeline), only a few of the 10 sessions included cultural-focused content, including discussions on how racial stress impacts health, parenting strategies for safety concerns, and considering family traditions of stress-coping and cultural activities. To assess the feasibility and acceptability of our intervention curriculum, including the minimal cultural resilience components, we collected qualitative and quantitative data from family participants. Qualitative data included interview questions focused on family’s perception of the utility and understanding of various intervention topics. Sample questions included “What would you have changed about the program,” “How understandable and “user friendly” was the program,” and “How enjoyable was talking about stress management and coping skills.” Quantitative data was collected through surveys completed by parents and teens that similarly targeted acceptability and comprehension of curriculum. The surveys also provided open-ended feedback for families to express additional feedback that they may not have been comfortable vocalizing directly to the facilitator.

**Table 1:** LEADS Development Timeline

Date	Event
Summer 2021	Cohort 1 begins (Partially Integrated Cultural Intervention vs. Health Education)
	Curriculum had very minimal focus on racial discrimination, cultural traditions Assessment of individual midpoint feedback (qualitative and quantitative) with each intervention family around Week 5 Midpoint qual and quant items focused on SMART goals, Fitbit concerns, Stress management exercises, General feedback (comprehension, enjoyment, difficulties), Text messages, Group/family communication Week 10 Group Testimonial (qualitative and quantitative) Qual & quant items focused on SMART goals, Fitbit concerns, PA and diet topics, Text messages, General feedback (comprehension, enjoyment, difficulties), **Stress Management exercises, Racial discrimination and health, Family and Cultural traditions for coping**
Fall 2021	Cohort 2 begins (Partially Integrated Cultural Intervention vs. Health Education)
	No changes to curriculum Assessment of individual feedback Week 3 and Week 7 with each intervention family Personalized individual family meetings for goal setting & Fitbit check-in Individual midpoint feedback (qualitative and quantitative) with each intervention family around Week 5 Midpoint qual and quant items focused on SMART goals, Fitbit concerns, Stress management exercises, General feedback (comprehension, enjoyment, difficulties), Text messages, Group/family communication Week 10 Group Testimonial (qualitative and quantitative) Qual & quant items focused on SMART goals, Fitbit concerns, PA and diet topics, Text messages, General feedback (comprehension, enjoyment, difficulties), **Stress Management exercises, Racial discrimination and health, Family and Cultural traditions for coping**
Spring 2022	Cohort 3 begins (Partially Integrated Cultural Intervention vs. Health Education)
	Individual feedback Week 3 and Week 7 with each intervention family Goal setting & Fitbit check-in Individual midpoint feedback (qualitative and quantitative) with each intervention family around Week 5 Midpoint qual and quant items focused on SMART goals, Fitbit concerns, Stress management exercises, General feedback (comprehension, enjoyment, difficulties), Text messages, Group/family communication Week 10 Group Testimonial (qualitative and quantitative) Qual & quant items focused on SMART goals, Fitbit concerns, PA and diet topics, Text messages, General feedback (comprehension, enjoyment, difficulties), **Stress Management exercises, Racial discrimination and health, Family and Cultural traditions for coping**
Fall 2022	Cohort 4 begins (Partially Integrated Cultural Intervention vs. Fully Integrated Cultural Intervention)
	Curriculum changes based on participant feedback from prior cohorts. Developed fully integrated cultural intervention to integrate cultural resilience into each session. Utilized more lay terms with self-affirmation and cognitive reframing. Created mission statement and positionality statements. Transparency of leadership and co-partnership with community Individual feedback Week 3, Week 5, and Week 7 with fully integrated group & partially integrated group Fully integrated cultural curriculum items focused on SMART goals, Fitbit concerns, Stress management exercises, Text messages, Group/family communication, **AND Racial Stress activities, Culture and Traditions** · Sought feedback on cultural resilience intervention components early into the program (Week 3) *Example items:* How have addressing racial stressors in our program been helpful? How has this been helpful? What about the conversations & activities have you enjoyed the most? Do you enjoy talking about racism and other barriers to health? What might you not enjoy about it? Are there any topics related to racial stress you think that we should cover in the future? What have you thought about the discussions and activities about culture and traditions? What have you thought about the discussions and activities about race and how you talk about race as a family? Partially Integrated Cultural Curriculum items focused on SMART goals, Fitbit concerns, Stress management exercises, Text messages, Group/family communication Week 10 Group Testimonial (qualitative and quantitative) Qual & quant items focused on SMART goals, Fitbit concerns, PA and diet topics, Text messages, General feedback (comprehension, enjoyment, difficulties), **Stress Management exercises, Racial discrimination and health, Family and Cultural traditions for coping, Racial Identity, Racial Socialization, Cultural Assets (Items focused on specific activities, e.g., family tree, healthy substitutes for cultural food)**
Sept-Dec 2022	Qualitative interviews conducted with prior LEADS families
Spring 2023	Cohort 5 begins (Partially Integrated Cultural Intervention vs. Fully Integrated Cultural Intervention)
	Curriculum changes based on participant feedback from prior cohorts, specifically to separate sessions covering health behavior goal setting/tracking and racial stress Individual midpoint feedback with fully integrated group & partially integrated group. Adjusted check-ins to be only at midpoint given participant feedback on burden of multiple feedback meetings Fully Integrated Cultural Curriculum items focused on SMART goals, Fitbit concerns, Stress management exercises, Text messages, Group/family communication, **AND Racial Stress activities, Culture and Traditions** *Example items:* How have addressing racial stressors in our program been helpful? How has this been helpful? What about the conversations & activities have you enjoyed the most? Do you enjoy talking about racism and other barriers to health? What might you not enjoy about it? Are there any topics related to racial stress you think that we should cover in the future? What have you thought about the discussions and activities about culture and traditions? What have you thought about the discussions and activities about race and how you talk about race as a family? Partially Integrated Cultural Curriculum items focused on SMART goals, Fitbit concerns, Stress management exercises, Text messages, Group/family communication Week 10 Group Testimonial (qualitative and quantitative) Qual & quant items focused on SMART goals, Fitbit concerns, PA and diet topics, Text messages, General feedback (comprehension, enjoyment, difficulties), **Stress Management exercises, Racial discrimination and health, Family and Cultural traditions for coping, Racial Identity, Racial Socialization, Cultural Assets (Items focused on specific activities, e.g., family tree, healthy substitutes for cultural food)**

While the majority of the qualitative and quantitative measures targeted health promotion and stress management curriculum, a few items targeted feedback on the few cultural-focused topics included in the session such as engaging in family cultural traditions and coping with racial discrimination. In reviewing the qualitative feedback, participants from Cohorts 1 through 3 endorsed acceptability of conversations about racism, discrimination and racial pride. One participant commented, *“I think when we have our little breakout sessions and we get the chance to talk candidly about, especially when we were talking about discrimination and race and everything, and how do that look from different perspectives. And, because sometimes the issue is no one is talking and no one is listening, you know, and that becomes a big problem because I have my own perspective but maybe if you hear how it make me feel, I might can change your perspective. Or, if you, if I’m able to express to you in words and not anger and not getting upset and not getting so emotional, but actually convey my sentiments of how I feel about what is happening in the world and to not even just me or my family but just people like me and my family and how that makes me feel.* Similarly, our quantitative feedback demonstrated high acceptability from families in our initial cohorts regarding topics of family cultural traditions and exploring coping techniques specific to racial stress (see Table S3 for data).

We also recognized families valued these culturally salient topics, as they consistently discussed concerns regarding community violence, racial stress, identity discussions, and complexities of African American parent–child dynamics during group sessions. However, our curriculum had very little emphasis on these topics that were prominent sources of stress to our African American families. Cultural needs are a crucial consideration in CBPR, and we realized that we needed to assess a greater degree of contextual stressors to better serve our families (Quattlebaum et al., 2021; Kipp et al., 2024). We were eager to adjust the curriculum to address the specific needs and concerns of African American families more effectively.

To fully understand how best to adapt our curriculum to be more culturally relevant and salient, we sought out additional feedback from our LEADS families that had already completed the program. Specifically, our research team conducted qualitative interviews with LEADS families to gather their recommendations on how to integrate activities and discussions related to racial stress and cultural and family resilience resources into the LEADS curriculum. In total, 16 adolescents and parents from LEADS participated in the interviews. Interview questions focused on exploring families’ personal experiences coping with racial stress and family discussions about racial stress, racial identity, and racial pride, as well as seeking out suggestions on specific activities on these topics to integrate into LEADS. Findings from these interviews showed that adolescents and parents endorsed a variety of cultural and family resilience resources for managing racial stress, including racial pride (parent and adolescent: 62.5%), egalitarian messages (e.g., equal treatment for all) (adolescent: 62.5%, parent: 50%), and preparing for racial bias (adolescent: 37.5%, parents: 50%). Adolescents and parents also endorsed several shared cultural and family activities, traditions, and routines to manage stress that could be integrated into the LEADS project, including using spirituality as a coping tool (parent: 25%), traditions around food (adolescent: 25%, parent: 12.5%), and structured family bonding time or activities (adolescent: 37.5%, parent: 50%). Some participants also suggested integrating preparation for bias messages and role playing how to handle a racial stressor into the LEADS project. These qualitative themes were critical in guiding our next steps in adapting our curriculum to integrate more culturally salient intervention curriculum activities.

In addition to leaning on qualitative feedback from LEADS families, our team also employed a range of strategies highlighted by Kreuter et al. (2003) to culturally adapt the LEADS curriculum. Specifically, we utilized evidential strategies (showing evidence relevant to African American populations) and sociocultural strategies (having discussions in context of cultural and social values) while adapting the curriculum. We responded to families’ engagement with social and cultural issues by facilitating more discussions about current events that specifically impacted African American communities, including community violence, racial stressors/discrimination and parenting, as well as addressing resources (Kreuter et al., 2003).

Additionally, we used culturally salient theoretical frameworks to guide our intervention changes. This included expanding our conceptual model (Figure S1 in supplemental materials) to incorporate Murry’s Integrated Stress Model for Black American Families and Utsey’s Africultural Coping theory, which highlights cultural differences, contextual risks related to racial stressors, and core protective cultural resilience resources unique to African American families (Murry et al., 2007, 2018). Drawing on the insights gained from our qualitative and quantitative findings, as well as leaning on these culturally salient theoretical frameworks, our Principal Investigators (Wilson, Martin) and intervention facilitators met weekly to thoroughly examine each facilitator guide to integrate culturally relevant content.

At this time we also developed a Mission Statement (see Table 2) and positionality statements (see Table S4 in Supplemental Materials). During the recruitment process for our initial cohorts, families expressed curiosity regarding why the target population exclusively focused on African American families. This highlighted the need for our research team to clearly communicate our values and intentions to the community. To address this, our research team developed a mission statement in co-partnership with our participating families that explicitly highlighted our team’s commitment to working with African American families (see Table 2). This helped align program values with the community's expectations and ensure that our team’s intentions were transparent and understood, fostering trust and engagement.

**Table 2:** LEADS Mission Statement

Our mission is to listen to the voice of Black parents and adolescents in understanding their daily lived experiences pertaining to stress, coping, and health. We have a strong focus on a holistic approach to faster family strengths and connectedness to improve overall health and wellbeing. The goal of our program is to use participatory approaches to co-create (equal partnership, equally valued participation) a family-based intervention to address cultural issues and stressors which have been linked to important health behaviours such as physical activity and diet.	

We integrated concepts from Murry and Utsey’s models (Murry et al., 2007, 2018; Utsey et al., 2007) as we revised the facilitator guides. These models purport core sources of cultural resilience that serve as a protective buffer of racial stress, including racial socialization, racial identity, and cultural assets. Racial socialization involves the explicit and implicit transmission of knowledge, values, and beliefs related to race, racism, and discrimination within a family unit (Neblett, 2023). It can include various practices such as cultural teachings, discussions about racial discrimination, promotion of cultural pride, preparation for bias encounters, and fostering cross-cultural relationships (Anderson & Stevenson, 2019). Racial socialization has the potential to promote positive racial attitudes, resilience, and a sense of cultural belonging in youth, while also equipping youth with the tools to navigate and challenge racial inequalities in society (Neblett, 2023). We integrated racial socialization into the LEADS program, including family discussions around what they are teaching their teens about what it means to be Black, creating a family tree to build understanding of traditions, and discussions regarding how parents prepare their teens for experiences of racism. By integrating racial socialization, our program addressed promoting positive racial attitudes, cultural belongingness, and a more comprehensive understanding of a community's strengths and resources.

Racial identity was another prominent cultural resilience resource that we integrated in LEADS. Racial identity refers to an individual's self-perception and understanding of their own racial background, the significance they attribute to it, and how they navigate and experience the world based on their racial identity (Neblett, 2023). Racial identity is a complex construct influenced by a variety of factors, including family, cultural, historical, and societal influences. It can evolve over time and may be influenced by individual experiences, social interactions, and broader socio-political contexts. Developing a positive and healthy racial identity is important for individuals as it can enhance self-esteem, resilience, and a sense of belonging, while also contributing to a more inclusive and equitable society (Neblett, 2023). This core cultural component was integrated into the LEADS program through activities such as practicing building a positive affirmation around one’s identity and identifying Black role models through family discussions.

Additionally, we integrated core cultural assets specific to African American families within our LEADS curriculum. Cultural assets refer to the positive attributes, values, and practices that are unique to a particular cultural or ethnic group (Neblett, 2023; Murry et al., 2007, 2018). These strengths can be manifest in various aspects of life, including family dynamics, social relationships, education, and overall well-being. Cultural strengths are often rooted in traditions, shared experiences, and historical resilience contexts. They can include characteristics such as strong community bonds, respect for elders, intergenerational support, collectivism, spirituality, adaptability, and a keen sense of identity and pride (Neblett, 2023). Recognizing and understanding cultural strengths is crucial for promoting cultural diversity, fostering inclusivity, and addressing disparities in various domains of society. With this framework, we integrated activities highlighting cultural strengths, including African dance, spirituality as a coping tool, and leaning on extended kinship and communalism for support.

Upon adapting our intervention curriculum to be more culturally salient, we were eager to hear from families on their response to the fully integrated cultural curriculum. Thus, we collected additional qualitative and quantitative data from LEADS families exposed to the adapted curriculum. We prioritized meeting with families at Week 3 of the program to gather feedback early into the intervention so that the material was fresh in their memory and to allow time to adjust any material later on in the intervention if needed. During the individual meetings with families, LEADS facilitators conducted brief semi-structured interviews asking for families’ perspectives on the intervention curriculum, with a particular emphasis on the culturally salient content. The quantitative feedback provided by families was collected through surveys that measured acceptability and usefulness of specific session activities, including culturally relevant content.

The qualitative and quantitative findings showed mixed results. During individual feedback interviews, several families reported that the delivery of our culturally tailored content came across unclear and somewhat difficult to relate to at times. With one participant reporting:

*“I didn’t understand how it [conversations about racial stress] was related to this type of health. It seemed to come out of thin air. It was odd.”* We also heard from families about the need to integrate language that highlighted African American families’ strengths as opposed to highlighting inequity and elevated risks, with a participant *noting "It feels weird when those questions are just thrown in there. It was weird to just be asked about what it’s like to be in a Black family. Like were you saying that Black families aren’t healthy? Or not able to be healthy?”.*

Although families reported the previously noted concerns, families also had positive feedback regarding the culturally salient curriculum. Specifically, one participant commented, *“I think it’s important because it teaches you in certain situations you can handle it one way or the other. And how do you let that drive you, and so I just using it as a driving force and say, you know what, I belong here. And so, you know, I don’t have to prove myself to them but in those situations you have to, because at work when you get promoted in order for you to be heard you have to be exceptional and that’s the only standard you have. And so I just think that is important in this program because that is a huge stressor.”* Furthermore, our quantitative feedback also demonstrated relatively high acceptability of these various topics (Table S3 see supplemental materials). Prominent activities and discussions endorsed were family communication about how to manage racial stress, managing community stressors (violence) and promoting community safety, spirituality as a way to cope with stress.

With this feedback, we re-examined each of our facilitator guides to address these concerns. Key content that utilized more deficits-based language was adjusted to emphasize the historical systemic inequalities that have contributed to exacerbated risks among African American populations. Additionally, we drew on a strengths-based lens to consider how African Americans lean on cultural traditions and values that are protective factors. Based on feedback that topics of health behavior goal setting and racial stress together were unclear, we presented this content in separate sessions rather than together. We also integrated activities recommended from prior LEADS families on how they recommended we integrate discussions related to racism and health and cultural strengths as part of our program.

After making these additional adjustments to our culturally relevant curriculum, we sought feedback from LEADS families exposed to this new curriculum. Both qualitative and quantitative findings showed improved acceptability of our culturally salient curriculum. Specifically, qualitative findings showed that cultural activities and discussions emphasized family traditions and values. One participant reported *“I love those activities because it makes me think back to different stuff like today it made me think about things my grandparents used to do when I was younger.”* Quantitative findings from cohorts 4 and 5 also demonstrated high acceptability of these adapted cultural resilience topics among both adolescents and parents (see Table 3).

**Table 3:** Cultural Resilience Intervention Acceptability Feedback – Cohorts 4 and 5

	Adolescent		Caregiver	
	M	SD	M	SD
*Utility of Cultural Resilience Tools*				
It was useful to identify cultural foods and cultural traditions around food	3.50	1.51	4.64	0.67
It was useful to build a positive self-statement about being Black	3.60	1.35	4.73	0.65
It was useful to discuss family communication about how to manage racial stress	4.00	1.05	4.45	0.82
It was useful to discuss family communication about cultural pride	3.70	1.57	4.45	0.69
It was useful to discuss spirituality as a way to cope with stress	4.20	0.79	4.73	0.47
It was useful to learn about the physical and mental health consequences of racism	3.90	1.29	4.64	0.67
*Overall Program Acceptability*				
The LEADS program has been useful for me	3.90	0.99	4.82	0.40
I enjoy the LEADS group sessions	4.00	0.67	4.82	0.40
I learned new things in the LEADS group sessions	4.10	0.88	4.73	0.65

Apriori goal for acceptability was ≥ 3.5 on a 1–5 scale

We also utilized our Essential Elements to guide the adaptations to our curriculum to ensure they aligned with our theoretical frameworks (Table S5 Supplemental Materials). Developing an essential elements and curriculum matrix that is sensitive to issues around racial stressors is crucial for creating effective programs that address the unique needs of African American populations. Racial stressors, such as discrimination, microaggressions, and systemic inequalities, can profoundly impact the mental and physical health of individuals within these communities (Neblett, 2023; Murry et al., 2007, 2018). By incorporating this understanding into the curriculum, program developers can provide targeted interventions and strategies that empower individuals to navigate and cope with these stressors effectively.

We also were intentional about considering the heaviness of discussions regarding racial stress and health inequities and the importance of presenting this information in a sensitive manner. Specifically, to account for this sensitive material (e.g., bullying, racial trauma and family stressors), at least one of the facilitators was a doctoral student in a Clinical Psychology program with thorough training in delivering culturally appropriate evidence-based mental health care. If any elevated mental health concerns were reported or noticed throughout group sessions, this facilitator followed up individually with the family to provide further support and families were referred to a local counseling clinic. All LEADS families were provided resources on local counseling clinics in the area, so they had tools to pursue more intensive support to manage stressors, including racial stress beyond the scope of the LEADS group intervention. Further, following discussions of race or racial stress, the LEADS team provided intervention families with an American Psychological Association (APA) pamphlet on racial stress and self-care. Throughout all sessions, the LEADS facilitators encouraged families to only share based on their comfort level to ensure no undue pressure was placed on families to discuss these challenging topics.

We were also intentional in reflecting the sensitive nature of the cultural resilience intervention components through our consent, assent, and protocol materials through the Institutional Review Board. Specifically, we highlighted our curriculum topics in detail within our study protocol. Additionally, within the risks and benefits section of our consent and assent, we clearly indicated that discussion of stress, race, and racial stress can be uncomfortable and may cause discomfort or distress. Thus, as part of the orientation and enrollment process of the LEADS project, families were well-informed on the range of topics covered and their rights as research participants to withdraw or no longer participate in the program.

#### Lesson 2: recruiting diverse research teams & engaging in self-reflection

Another prominent lesson learned throughout LEADS was the need to cultivate a diverse research team that continually engaged in self-reflection. To ensure families felt safer, we recognized the importance of recruiting more research team members of color who may share similar lived experiences. This lesson reflects NBLIC’s CBPR principle that understanding the diversity within African American families requires specific strategies. One such strategy involved training our research team to comprehend the African American experience better. This strategy also aligns with Kreuter’s (2003) emphasis on constituent involving strategies (having researchers of the same lived experiences of participants). We crafted recruitment efforts to specifically look for research assistants of color through courses being offered at our university. Specifically, we presented information about our project to undergraduates in a Psychology of the African American experience class taught by a member of our research team (Martin). We also recognized the need for self-reflection as a diverse team. Thus, we tasked all research team members to create positionality statements. These statements required our team to reflect on their biases and address them to honor the families we serve. This led our research team to reflect on how to engage with African American families in a way that is meaningful for them.

Building upon the insights from Kreuter’s (2003) work, we proactively took a step forward by organizing weekly research workshops for our research team. These workshops aimed to enhance our understanding of cultural nuances, increase cultural sensitivity, and ensure that our research practices were aligned with the needs and values of the communities we served. We also engaged in reflexivity journaling so students could unlearn any biases or stereotypes they held toward African American families. As part of their journaling process, students were tasked with reflecting on the workshops, particularly focusing on the session that explored the origins of these biases and stereotypes rooted in pseudoscience. This training allowed us as a research team to understand how our biases shaped the way we interacted with the research. For our non-African American team members, we made sure they understood how to interact with families in a way that was not stigmatizing or threatening. To combat this, we had team members read literature from African American psychologists about African Americans to understand the writing styles and what it looks like to research African Americans from a strength-based perspective. Additionally, we had team members reflect on ways they have moved through their biases in the research. This proved to be important, especially during recruitment events in the community with more families engaging in screenings and consenting to participate in our ongoing study.

#### Lesson 3: recognizing heterogeneity

We also learned throughout the LEADS Project the need to recognize and celebrate the heterogeneity of African American families given that need for a heterogenous lens when examining racial stress, protective buffers, and long-term outcomes (Clark et al., 1999; Volpe et al., 2022). There was notable diversity across the LEADS families including adolescent age and sex. Among LEADS adolescents, 60.6% were female and had an average age of 13.8 (*SD* 2.06), specifically ~ 58% of youth were aged 11–14 years old and ~ 43% were aged 15–17 years old. Among our distinct families, we learned during sessions in which we discussed family’s racial socialization practices (talking to family about race related experiences and values) that teens were having vastly different experiences from each other. Some younger adolescents (ranging from 11 to 14 years old) reported having little or no experiences of discrimination in building their racial identity. Specifically, an 11-year-old teen reported that he/she thought little about race and did not notice skin color. Conversely, many older teens (ranging from 15 to 17 years old) had a more fully formed understanding of their experiences of discrimination and racial identity. For example, a 17-year-old teen participated in a Black History Month planning committee at his/her high school and found this to be a source of cultural pride. This teen had a strong understanding of microaggressions and other forms of discrimination through personal experiences and experiences at school. Given these differing experiences among our LEADS teens, we learned the importance of recognizing heterogeneity across families and adjusting our discussions as needed based on these differences.

Among LEADS parents and caregivers, we also saw variability regarding their willingness to openly discuss racial stressors given the diversity in lived experiences (Clark et al., 1999; Volpe et al., 2022). Based on caregiver reports and existing research, this was predominantly due to many factors like age, ethnicity, family makeup, and birthplace (north v. south), and history of cultural experiences. It was a learning curve for our research team to understand how to make sure parents felt heard and supported enough to share their experiences. We were able to step back and allow our participants to have conversations and make the connections on their own. Our biggest lesson here was creating and sustaining a community participatory approach to allow our participants to draw conclusions from the information given to them that made the most sense for their family. Specifically, it was important to consider the cultural strengths, family traditions, and other aspects of the community context. By acknowledging and incorporating these important aspects of community life into the intervention process, community-based research can be more effective and culturally responsive. This approach can also help ensure that the research is grounded in the community's experiences, needs, and values, leading to more meaningful and relevant intervention strategies that can benefit the community (Wilson & Sweeney, 2024).

#### Lesson 4: utilizing strengths-based research approaches

Strength-based research in CBPR emphasizes the exploration and recognition of existing strengths and assets within a community (Merves et al., 2015). This approach shifts the focus from solely identifying problems and deficits to understanding and leveraging the positive aspects and resources within the community (Neblett, 2023; Murry et al., 2018). With this approach, researchers collaborate with community members to identify and build upon the inherent strengths, skills, knowledge, and capacities that exist within the community (Wilson & Sweeney, 2024). This approach recognizes that communities possess valuable resources and expertise that can contribute to their own growth and development. By emphasizing strengths, CBPR promotes empowerment, resilience, and self-determination among community members, fostering a sense of ownership and pride in their community's abilities and potential (Israel et al., 1998). This approach not only leads to more effective and sustainable interventions (Cyril et al., 2015; Wallerstein & Duran, 2010) but also fosters a positive and asset-based narrative that challenges stigmatization and promotes a more holistic understanding of communities and their capacities.

Throughout our 2-years of the LEADS Project, the program underwent significant changes, and one of the most impactful was transitioning to a strength-based approach in our curriculum. This transition involved incorporating African American representation and feedback from African American facilitators and integrating in discussions on cultural strengths, racial identity, and family racial socialization practices in our sessions. This task proved to be complex because we realized how much we needed to grow in our strength-based approaches. These changes were guided by listening to our families’ qualitative feedback and soliciting feedback from our African American intervention facilitators on what could be improved to make our curriculum more strength-based in focus (Neblett, 2023). Specifically, one of the primary concerns vocalized by families during individual feedback interviews with our facilitators was how they felt their lived experiences were not captured by how the health information we presented with statistics of African Americans health risks and deficits being emphasized over strengths.

To address this concern, we reviewed every week of our curriculum to adjust the language regarding health data that was negatively worded or did not show the full experience of African American families. Specifically, this included adjusting any deficit-based health information that showed negative health outcomes to integrate the social or political context. This was important to ensure that participants felt a sense of empowerment moving forward with their health promotion goals rather than defeat or stigma. Additionally, changes included having more discussions in sessions about cultural strengths and how those can be reflected in our health. Changes also included in-depth discussion about racial identity and families’ racial-ethnic socialization practices, which are recognized as strengths-based protective factors (Neblett, 2023). Further, we incorporated depictions of African American figures as models for strength-based efforts in Black families. We focused on challenging deficit-based narratives and promoting a more accurate understanding of the strengths and resilience within Black and African American families (McAdoo, 2007). McAdoo (2007) specifically highlighted the significance of family support networks and coping strategies that Black and African American families employ to navigate and overcome adversity. McAdoo (2007) stressed the importance of extended family, kinship networks, and community connections in providing emotional, social, and material support to individuals and families. Recognizing and leveraging these support networks can be crucial for strengthening Black and African American families and promoting positive outcomes. McAdoo (2007) also argued that Black and African American families have a long history of resilience, often drawing from cultural values, spirituality, and community resources to thrive in the face of challenges. Recognizing and fostering these resilience factors can help Black and African American families build upon their existing strengths and navigate systemic barriers effectively.

Overall, by adopting a strength-based perspective, researchers and practitioners can develop interventions that empower and support Black and African American families in achieving their full potential. Utilizing a strength-based focused we refocused our framework to reflect positive outcomes in African American communities, integrating cultural strengths into our curriculum, and facilitating discussions that included cultural strengths (e.g., religiosity, family traditions, cultural history). This pushed us to sustain a curriculum that was strength-based, presenting information that made families feel a sense of pride and competence.

### Implications and recommendations from lessons learned

#### Need for incorporating community collaboration for program tailoring

Throughout the evolution of the LEADS project, we recognized the value of community participatory research to guide changes and respond to lessons learned. Community collaboration is vital in tailoring a program to the specific needs and concerns of the target population. Community members have firsthand knowledge and lived experience that can shape the design, content, and delivery of the program. Involving community leaders, organizations, and community partners in the development process ensures that the program is culturally relevant, respectful, and aligned with the values and priorities of the community (Wilson & Sweeney, 2024). This collaborative approach also fosters a sense of ownership and engagement, as community members can see themselves reflected in the program, leading to increased participation and retention.

Furthermore, community collaboration helps identify community-specific resources, strengths, and assets that can be integrated into the program. By acknowledging and building upon existing community resilience and support networks, the program becomes more effective and sustainable. Community collaboration helps in identifying potential barriers or challenges that might hinder engagement or implementation, allowing for proactive strategies to be developed and implemented (Glasgow et al., 1999). Involving the community in program tailoring ensures that it is responsive to the community’s needs, and aspirations, resulting in a program that is more likely to achieve positive outcomes and impact the well-being of the target population.

#### Importance of research team diversity and self-reflection

Another key recommendation we propose is the importance of building a diverse research team with equal participation. Cultivating a diverse research team with members that share lived experiences with the target study population is of utmost importance. It brings valuable perspectives, insights, and cultural competence that are essential for understanding the nuances and complexities of the communities being studied. When the members of a research team share diverse backgrounds, it fosters a sense of trust, relatability, and mutual understanding. Diverse research teams can also build trust and comfortability among participants from marginalized communities, such as African American populations, which may enhance engagement, participation, and retention. This collaborative approach allows for the identification of potential biases, challenges, and opportunities that may arise during the research study. It also ensures that different viewpoints are considered, enabling the development of culturally sensitive methodologies, recruitment strategies, and data analysis techniques that accurately capture the experiences and realities of African American individuals.

In addition, collaboration within a diverse research team fosters a culture of inclusion and respect. It promotes the sharing of knowledge, expertise, and best practices, which can lead to innovative approaches and more impactful research outcomes. By valuing the contributions of team members with diverse backgrounds and lived experiences, researchers can create an environment that encourages open dialogue, critical thinking, and creativity. There must be extra precautions taken so that there is not any undue emotional burden on team members of color. This includes blind team-wide reviews, de-centralizing team structures, and creating an open environment as you would with community members. This collaborative synergy can lead to research that is more comprehensive, meaningful, and applicable to marginalized populations, as well as contribute to a broader understanding of health and well-being in diverse communities.

Within a diverse research team working with families from minority racial-ethnic backgrounds, it is imperative that team members challenge and educate themselves about negative stereotypes that may exist about their target population. Researchers should actively work to dispel the biases and assumptions that have historically influenced research and perpetuated negative narratives. This involves critically examining one's own biases and preconceptions and actively seeking out diverse perspectives within the target community. By doing so, researchers can better understand and appreciate the rich diversity, strengths, and talents that exist within the community.

#### Need for examining heterogeneity in African Americans

An important lesson learned from LEADS was understanding the heterogeneity of the participating community. This may involve understanding differences in gender, age, and identity that uniquely shape African American experiences. When conducting studies involving African American populations, it is crucial to be sensitive to sample heterogeneity to increase engagement and retention. To achieve this, it is important to adopt culturally sensitive approaches and actively involve community leaders and participants in the research process. This involves acknowledging historical and current disparities in research, addressing any concerns or fears, and ensuring that the study design is respectful of the cultural values and beliefs of the African American population. In summary, researchers should invest time in building trust and rapport with the community by engaging in open and transparent communication.

Secondly, it is essential to actively recruit and involve African American individuals in all stages of the research process. This can be done by partnering with community-based organizations, religious institutions, and healthcare providers who have established relationships with the target population. Involving community leaders as advocates and research partners can help build credibility and increase participation.

Another way to think about how to understand the heterogeneity of participants is to look at intersectionality theory. Intersectionality involves the study of the ways that race, gender, disability, sexuality, class, age, and other social categories are mutually shaped and interrelated through forces such as colonialism, neoliberalism, geopolitics, and cultural configurations to produce shifting relations of power and oppression (Rice et al., 2019). Intersectionality is designed for us to understand that everyone has multiple identities that shape their worldview. We can use intersectionality to help us understand the diversity in experiences as we aim to have a heterogeneous view of African American populations. There are differences in cultural barriers, cultural traditions, strengths, and the histories of a broad community, but also differences in identities that lead to different experiences. Some identities to consider include gender, socioeconomic status, birthplace, family makeup, sexuality, and religion. By recognizing and respecting these diverse aspects, we can better address individual needs.

#### Importance of strength-based health promotion efforts

In line with Murry’s integrated model, as well as work by other leading researchers in the field, cultural resilience resources are culturally specific protective factors that can build individual and family capacity to rebound and adapt in response to extreme stress and adversity, including racial stress (Murry et al., 2018; Woods-Jaeger et al., 2020; Sweeney et al., 2023; Wilson et al., 2015, 2022). Growing evidence demonstrates the protective nature of cultural resilience resources, with studies showing positive health and well-being outcomes associated with these resources (Cooper & Mcloyd, 2011; Neblett et al., 2009; Quattlebaum et al., 2024). According to extant literature, cultural assets, ethnic-racial socialization competency, and ethnic-racial identity are recognized as prominent sources of cultural resilience. Though these constructs are often considered to be distinct from each other, there is evidence to suggest that there is often a dynamic, interactive process between these constructs that effectively buffers chronic and racial stress and supports positive outcomes (Gaylord-Harden et al., 2012). Moreover, leveraging the unique cultural resilience resources developed within African American families to be integrated into family-based interventions has been a growing focus in recent trials and needs to be implemented to understand the lived experience of African American families (Anderson et al., 2018; Brody et al., 2006). A strength-based approach requires active collaboration and partnership with African American communities. By involving the community as active participants and experts in their own experiences, researchers can ensure that the research is culturally relevant, respectful, and impactful.

Strength-based approaches emphasize resilience and positive narratives within African American research. Researchers should actively seek out and highlight stories of success, triumph, and resilience within the community. This can involve documenting and sharing personal narratives, achievements, and strategies that African Americans have employed to overcome challenges and achieve positive outcomes. By amplifying these stories, researchers can contribute to a more balanced and accurate representation of African American experiences, fostering pride, hope, and empowerment within the community and beyond.

## Conclusions

In conclusion, our team developed the LEADS intervention to specifically address racial stressors, which should be a key consideration for health promotion programs seeking to improve positive health outcomes and that promote health equity among ethnic-racial minorities. The development and lessons learned described in this paper provide important insights for using a participatory process, including developing a mission statement and process for integrating the voice of community participants throughout the process. Further research is needed to demonstrate the efficacy of conducting strength-based programs to address racial stressors and improve positive health outcomes among African American families and communities.

## Supplementary Information

Below is the link to the electronic supplementary material.

Supplementary file1 (DOCX 163 KB)

## Data Availability

N/A.

## Code availability

N/A.
