# Supplementary material for: Strength-based strategies for addressing racial stressors in African American families: lessons learned from developing the LEADS health promotion intervention
Source: J Behav Med. 2024 Aug 10;47(6):951–64. doi: 10.1007/s10865-024-00509-y (PMC11499371; doi:10.1007/s10865-024-00509-y)
Supplement: Supplementary file 1 — Supplementary file1 (DOCX 163 KB) [file 10865_2024_509_MOESM1_ESM.docx]

**Supplemental Files**

**Table 1S.** Demographics

| Adolescent sex (% female) | 60.6 |
| --- | --- |
| Adolescent Age (years) *M* (*SD*) | 13.8 (2.06) |
| Adolescent BMI %ile *M* (*SD*) | 97.2 (2.69) |
| Parent sex (% female) | 97.2 |
| Parent Age (years) (*M*, *SD*) | 45.5 (10.3) |
| Parent BMI (kg/m^2^) (*M*, *SD*) |  |
| Marital Status (%) |  |
| Married | 19.4 |
| Divorced | 27.8 |
| Never Married | 36.1 |
| Parent Education (%) |  |
| High school graduate | 8.3 |
| Some college but no degree | 27.8 |
| Associate degree in college | 19.4 |
| 4 Year College | 19.4 |
| Graduate training or professional degree | 25 |
| Parent Annual Household Income (%) |  |
| Less than $10,000 | 8.3 |
| $10,000-$19,999 | 8.3 |
| $20,000-$29,000 | 16.7 |
| $30,000-$39,999 | 25.0 |
| $40,000-$49,999 | 13.9 |
| $50,000-$59,999 | 13.9 |
| $70,000-$79,999 | 5.6 |
| $90,000-$99,999 | 2.8 |
| $100,000-$149,999 | 5.6 |

**Table S2.** Partially and Fully Integrated Curriculum Comparison

| **Week** | **Partially Integrated Cultural-Focused Curriculum**  **(Summer 2021-Spring 2022)** | **Fully Integrated Cultural-Focused Curriculum**  **(Fall 2022-Spring 2023)** |
| --- | --- | --- |
| 1 | Topic: Introduction   - Provide overview of program and review stress management - Positive communication skills - Group ground rules - Stress coping exercise: Deep Breathing - Family Bonding activity: Plan family activity | Topic: Introduction & Mission Statement   - **Principal Investigators meet & greet and group discussion on mission statement** - **Provide overview of program and review stress management, including group discussion on African American health inequities, stress and health, cultural strengths for coping** - Positive communication skills - Group ground rules - Stress coping exercise: Deep Breathing - **Family Bonding activity: Plan family activity. Identify weekly stressors, including racial stress** |
| 2 | Topic: Stress and Tracking for Health Behaviors   - Identify stressors and impacts on health - Review self-monitoring - Stress coping exercise: Body Scan - Family Bonding activity: Choose tracking tool | Topic: Tracking & Goal Setting for Health Behaviors   - Review self-monitoring and goal setting (SMART goals) - Stress coping exercise: Body Scan - Family Bonding activity: Choose tracking tool |
| 3 | Topic: Goal Setting, Cognitive Reframing, Positive self-statement   - Review goal setting (SMART Goals) - Exercise as a stress management - Positive self-statement - Stress coping exercise: Cognitive reframing - Family Bonding activity: Set family related health behavior goal | Topic: Stress, Cognitive Reframing, Positive self-statement   - **Identify stressors unique to families, including racial stress, and impacts on health** - **Stress coping exercise: Cognitive reframing, including using cultural coping for racial stress** - **Trailer for Hate U Give movie and discussion on racial coping** - **Positive self-statement specific to one’s Black identity pride** - **African dance** - **Family Bonding activity: Check in on goal setting as a family, identify Black role model, family discussion on stressors** |
| 4 | Topic: Life Balance   - Lifestyle approach to health behavior changes, caloric energy in/energy out - Barriers to life balance - Stress coping exercise: Guided Imagery - Family Bonding activity: Develop family routine plan/calendar | Topic: Physical Activity   - National guidelines for physical activity - Solutions to PA barriers and how exercise can be used as a stress coping strategy - **Culturally relevant exercise (line dancing) and barriers (hairstyles)** - Parent descriptive praise, support, providing choice (“escape hatch/volition/choice”) - **Descriptive praise specific to Black identity** - **Black history of yoga and spiritual practice** - Stress coping exercise: Beginner Yoga - **Family Bonding activity: Family culturally relevant physical activity (dancing)** |
| 5 | Topic: Eating Behaviors   - Portion sizes, hunger and satiety cues, mindful eating, family-based meal preparation - Emotional eating - Stress coping exercise: Mindful Eating - Family Bonding activity: Prepare a healthy meal as a family | Topic: Eating Behaviors   - Portion sizes, hunger and satiety cues, mindful eating, family-based meal preparation - **Emotional eating and related cultural chronic stressors** - **Cultural foods, recipes (USDA African American recipes), and traditions** - **Healthy tweaks to cultural foods (cultural recipes) and cultural events (cookouts, holidays)** - Stress coping exercise: Mindful Eating - **Family Bonding activity: Prepare a healthy cultural meal as a family** |
| 6 | Topic: Physical Activity   - National guidelines for physical activity - Solutions to PA barriers and how exercise can be used as a stress coping strategy - **Spirituality for coping** - Parent descriptive praise, support, providing choice (“escape hatch/volition/choice”) - Stress coping exercise: Beginner Yoga - Family Bonding activity: Family physical activity | Topic: Life Balance   - Lifestyle approach to health behavior changes, caloric energy in/energy out - **Barriers to life balance, including contextual factors (community violence)** - **Cultural strategies to build life balance (spirituality, positive self-statements) and create family routines (prayer before meals)** - Stress coping exercise: Guided Imagery - **Family Bonding activity: Develop family routine plan/calendar, considering cultural routines** |
| 7 | Topic: Racial stress, health consequences, parenting, coping, support for health behaviors   - **Racial discrimination and health/well-being consequences** - **Parenting strategies from a cultural context (safety concerns)** - Supporting teens with health behavior goals (autonomy support strategies, “You provide, they decide”), helping teens ask for support with health behaviors - Strategies for managing difficult family conversations - **Coping strategies specific to racial stress (journaling, community support, mental health resources, etc.)** - Stress coping exercise: 5 Senses Mindfulness Activity - Family Bonding activity: Practice family communication tools for reaching goals | Topic: Racial stress, health consequences, parenting, coping, support for health behaviors   - **Racial discrimination and health/well-being consequences** - **Family conversations about racial stress and racial identity** - **Parenting strategies from a cultural context (safety concerns)** - Supporting teens with health behavior goals (autonomy support strategies, “You provide, they decide”), helping teens ask for support with health behaviors - **Strategies for managing difficult family conversations, including racial stress** - **Coping strategies specific to racial stress (journaling, community support, mental health resources, etc.)** - Stress coping exercise: 5 Senses Mindfulness Activity - **Family Bonding activity: Journaling for managing racial stress** |
| 8 | Topic: Sedentary Behaviors & Screens   - **Health risks for sedentary behavior, screen time use among African Americans** - Substituting healthy alternatives to sedentary behavior, screen time, and junk food - Parent skills: You provide, they decide around screen time; limit setting, monitoring - Stress coping exercise: Progressive Muscle Relaxation - Family Bonding activity: Make a family screen time budget | Topic: Sedentary Behaviors & Screens   - **Health risks for sedentary behavior, screen time use among African Americans** - **Substituting healthy alternatives to sedentary behavior, screen time, and junk food with cultural activities (African American social dancing)** - **Exposure to racial stress online** - Parent skills: You provide, they decide around screen time; limit setting, monitoring - Stress coping exercise: Progressive Muscle Relaxation - **Family Bonding activity: Make a family screen time budget, Brainstorm family alternatives to screen time, including cultural family activities** |
| 9 | Topic: Family Tree & Positive Communication   - **Family Tree of racial identity beliefs, cultural traditions, stress/coping patterns** - **Active listening and push-pull language,** **using these skills during difficult family conversations about racial stress and coping** - Stress coping exercise: Intention Setting Meditation - **Family Bonding activity: Pick family tradition from family tree, brainstorm continuing or improving family traditions** | Topic: Family Tree, Positive Communication, Racial Socialization   - **Family Tree of racial identity beliefs, cultural traditions, stress/coping patterns** - **Active listening and push-pull language, using these skills during difficult family conversations about racial stress and coping, learning from family storytellers** - **Racial socialization discussion, transgenerational messages of cultural pride** - Stress coping exercise: Intention Setting Meditation - **Family Bonding activity: Pick family tradition from family tree, tell a story of family history, brainstorm continuing or improving family traditions** |
| 10 | Topic: Review & Testimonial   - Review topics and stress coping strategies - Relapse prevention, planning for high-risk situations (discuss future stressors) - Create stress coping/health goal setting toolbox - Testimonials | Topic: Review & Testimonial   - Review topics and stress coping strategies - Relapse prevention, planning for high-risk situations (discuss future stressors) - Create stress coping/health goal setting toolbox - **Testimonials, including curriculum on racial stress, coping, health, culture, and socialization** |

**Table S3.** Cultural Resilience Intervention Acceptability Feedback – Cohorts 1 through 3 (n =15)

|  | **Adolescent** | | **Caregiver** | |
| --- | --- | --- | --- | --- |
|  | **M** | **SD** | **M** | **SD** |
| **Utility of Cultural Resilience Tools** |  |  |  |  |
| Talking about racial discrimination and coping was useful for me. | 4.29 | 0.95 | 4.5 | 1.07 |
| I enjoyed talking about family cultural traditions. | 4.14 | 0.90 | 4.62 | 0.74 |
| **Overall Program Acceptabilty** |  |  |  |  |
| The LEADS program has been useful for me | 4.14 | 0.90 | 4.37 | 0.74 |
| I enjoy the LEADS group sessions | 3.71 | 1.25 | 4.75 | 0.70 |
| I learned new things in the LEADS group sessions | 4.29 | 0.75 | 4.25 | 0.70 |
| The LEADS program has been easy for me to understand | 4.00 | 1.00 | 4.62 | 0.74 |
| I would recommend the program to other families I know. | 4.43 | 0.53 | 4.62 | 0.74 |
| Since the LEADS program, I am more supportive of my family. | 4.14 | 1.07 | 4.50 | 0.53 |

**Note**: Apriori goal for acceptability was >3.5 on a 1-5 scale.

**Table S4**. LEADS Principal Investigators Positionality Statements


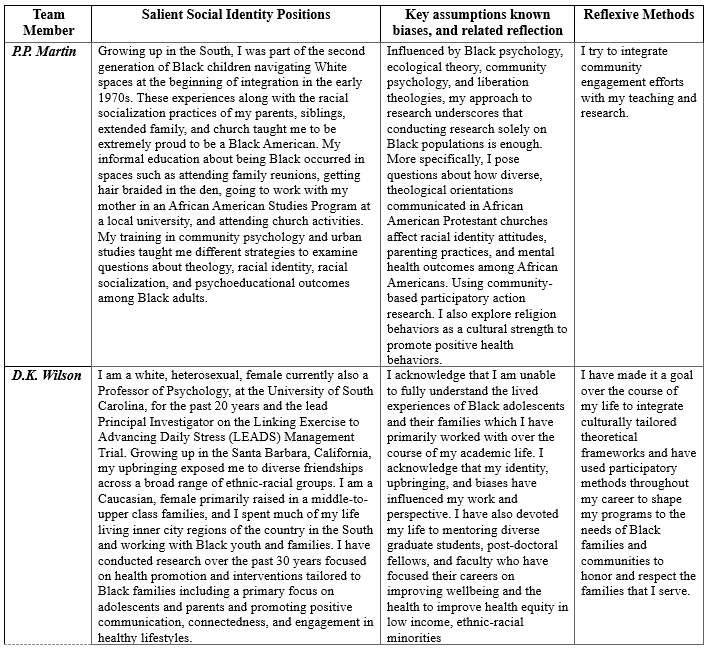


**Table S5.** Example Intervention Essential Elements & Program Elements

| **Theory** | **Essential Elements** | **Description of Program Elements** |
| --- | --- | --- |
| Social Cognitive Theory  & Family Systems Theory | Social Support | Use strategies for eliciting and providing social support for stress management and health behaviors from parents. |
| Family Systems Theory, **Cultural Tailoring**,  & **Cultural Assets** | Communication Skills & Autonomy Support | **Use positive communication (reflective listening, problem-solving, shared decision-making) to increase family bonding**, reduce stress, and improve health behaviors. |
| Social Cognitive Theory  & Resilience Theory | Goal Setting, Family Routines, & Self-Regulation | Set family goals to reduce stress and improve health behaviors. Structured daily family routines and time together to improve self-regulation (emotional, behavioral) through positive reinforcement. |
| Social Cognitive Theory | Group-based Behavioral Skills & Connectedness | Share anticipated or actual barriers to physical activity and problem-solve. Select a weekly collective group-based physical activity goal (e.g., Fitbit tracking). |
| **Cultural Tailoring**, **Cultural Assets**,  & Family Systems Theory | Cultural Assets & Strengths-Based Values | **Communicate about ethnic-racial identity and foster cultural pride and self-esteem.** Develop proactive coping strategies to address racism and chronic stress. Learn interactive behavioral skills to reduce stress (spirituality, meditation, mindfulness). |
| **Cultural Tailoring**, **Cultural Assets**, Family Systems Theory,  & Social Cognitive Theory | Cohesion & Mastery of Culture, Behavior, Emotions | **Address social and cultural topics, including family cohesion, spirituality, collectivism, life balance, cultural traditions** (e.g., cooking), and cognitive reframing of negative emotions. |

**Figure S1.** LEADS Intervention Conceptual Framework


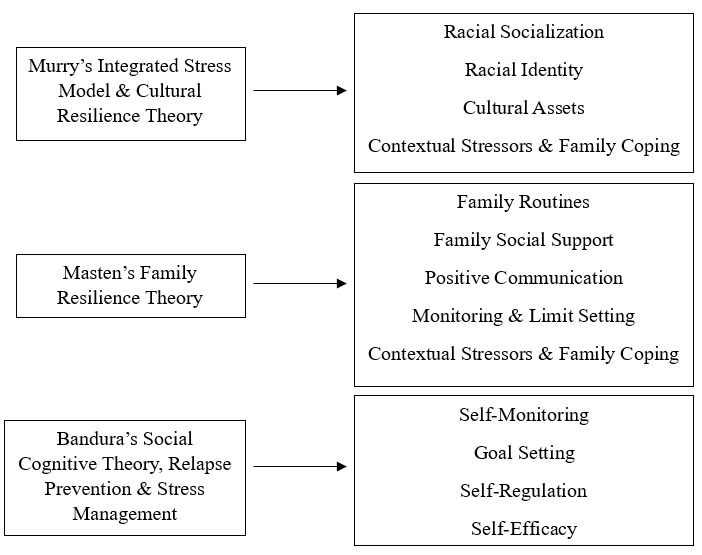


**Note.** Conceptual framework leans on insights from Murry et al., 2018, Masten & Monn, 2015, and Bandura, 2001
